# General practitioners’ knowledge, attitudes, beliefs and practices surrounding the prescription of e-cigarettes for smoking cessation: a mixed-methods systematic review

**DOI:** 10.1186/s12889-022-14696-3

**Published:** 2022-12-23

**Authors:** Melis Selamoglu, Bircan Erbas, Karthika Kasiviswanathan, Chris Barton

**Affiliations:** 1 Department of General Practice, School of Public Health and Preventive Medicine, Monash University, Melbourne, Australia; 2 Level 5, 553 St Kilda Road, Prahan, VIC 3181 Australia; 3 Department of Public Health, School of Psychology and Public Health, La Trobe University, Melbourne, Australia; 4 School of Public Health and Preventive Medicine, Monash University, Melbourne, Australia

**Keywords:** knowledge, attitudes, e-cigarette, smoking cessation, general practice, systematic review

## Abstract

**Background:**

General practitioners (GPs) play an important role in providing patients who smoke with health information, support and treatment to encourage them to quit smoking. Despite conflicting evidence on the effectiveness of electronic cigarettes (e-cigarettes) as a smoking cessation aid, there is growing interest in the role e-cigarettes might play as an alternative to smoking tobacco. This systematic review aims to synthesise evidence from qualitative, quantitative and mixed-methods studies of the knowledge, attitudes, beliefs and social norms of GPs with respect to the use of e-cigarettes as smoking cessation aids.

**Methods:**

This study adhered to the PRISMA guidelines. Studies from MEDLINE, CINAHL, SCOPUS, PsycINFO, EMBASE and grey literature were searched. Two independent reviewers screened abstracts and full-text articles to identify studies that met the inclusion criteria. A data extraction form was used to extract relevant data from included papers and were quality appraised using the MMAT checklist. A PRISMA flow diagram was used to record the flow of papers and reasons for exclusion. Studies were included if they collected quantitative, qualitative or mixed methods data to determine knowledge, attitudes, beliefs and social norms of GPs for use of e-cigarettes as smoking cessation aids.

**Results:**

A total of 4056 abstracts were screened and 25 articles were included. Our findings showed that GPs had mixed views on recommending e-cigarettes as a smoking cessation aid. Some GPs were optimistic and had recommended e-cigarettes to their patients. Others were reluctant and disagreed that e-cigarettes are an effective method to quit smoking. Most GPs lacked knowledge and confidence in having discussions with patients around e-cigarette safety and efficacy as smoking cessation alternatives.

**Conclusion:**

This systematic review shows there are mixed views on e-cigarettes as smoking cessation aids. Clear guidance on the role of e-cigarettes is needed to inform and upskill GPs about e-cigarettes for smoking cessation.

**PROSPERO registration:**

CRD42021227612.

**Supplementary Information:**

The online version contains supplementary material available at 10.1186/s12889-022-14696-3.

## Introduction

There are 1.1 billion adult smokers around the world and over half (60%) of them want to quit [1]. General practitioners (GPs) play a crucial role in providing patients who smoke with health information, support and treatment to quit smoking. Moreover, they are often the first point of contact for patients who seek information about smoking cessation. Brief and simple advice from a GP can help smokers take the initial steps necessary to quit smoking and support them with quit attempts [2].

In the past decade there has been a surge in the use of electronic cigarettes (e-cigarettes) /ENDS (electronic nicotine delivery system) in the community. These devices are battery-powered devices heat liquids (e-liquid) to produce nicotine and/or other substances through an aerosol which are inhaled by the user, also known as ‘vaping’ [3]. E-cigarettes are available in two formats, refillable open systems (tank, mods, vape pens) and closed systems (pod-based, pod mod, disposable) [4]. The e-liquid in e-cigarettes contain nicotine, propylene glycol, glycerine, flavouring agents and other chemicals [4].

It is estimated that the use of e-cigarettes has substantially increased from 35 million users in 2015 to 68 million users in 2020 [5, 6]. Prevalence rates have also increased with daily e-cigarette use among current smokers in Australia increasing from 1.5% in 2016 to 3.2% in 2019 [3]. Furthermore, an increase of 6.3% in 2020 to 7.1% in 2021 was reported in the UK among adults using e-cigarettes [7].

Little is known about GPs’ preparedness to have discussions with their patients and their intentions to prescribe e-cigarettes as a smoking cessation aid. Therefore, it is timely to synthesise the current literature describing the knowledge, attitudes, beliefs and social norms of GPs with respect to e-cigarettes as smoking cessation aids.

## Methods

This review adhered to the Preferred Reporting Items for Systematic Review and Meta-Analysis (PRISMA) guidelines [8] and the study protocol was registered on PROSPERO (CRD42021227612). A detailed description of the methods used in this review can be found in the protocol paper published elsewhere [9]. The methods described below are a summary.

### Data sources and search strategy

MEDLINE, CINAHL, EMBASE, SCOPUS and PsycINFO databases were searched for studies reporting on knowledge, attitude, social norms and perceived behavioural control of GPs (defined as primary care doctors, family physicians or their equivalent) and the use of e-cigarettes, or vaping, as a smoking cessation aid.

Moreover, the first ten pages of results of a Google search were screened to identify additional relevant peer reviewed or grey literature. Hand-searching of the reference lists of the included studies was undertaken to identify relevant articles that may not have been identified through database searches.

### Eligibility screening

Articles reporting qualitative, quantitative or mixed method studies that met the inclusion criteria were included. Articles that were in a language other than English, reviews or editorials, letters, commentary and opinion or perspective pieces, conference proceedings, protocol papers, systematic reviews and abstracts without full text were excluded.

### Quality appraisal and risk of bias assessment

The Mixed Methods Appraisal Tool (MMAT) [10, 11] checklist was used to assess the quality and risk of bias of the qualitative, quantitative and mixed methods articles that were included. Two authors (MS and KK) independently assessed the quality of studies as high, medium or low. The quality of studies can be found in the Supplementary file (S1). Articles were not excluded based on their rating but the ratings were used to guide the interpretation of findings and weight given to findings from studies within the synthesis.

### Data extraction and synthesis

A data extraction form was used to extract relevant data from the included studies. Data was extracted by the first author (MS) and a second author (CB) independently extracted data from the first 20% of articles to check accuracy. The details of study characteristics included; publication details (year of publication, author, title journal of publication etc), location of study and sample size. We further recorded the type of data collected (qualitative, quantitative, mixed method), aims/purpose, study design, framework/theory (if applicable) and key findings. The outcome and intervention measures included; GPs knowledge, attitude and beliefs, GPs recommendation of e-cigarettes, GPs intentions to prescribe e-cigarettes and the knowledge and support of regulations/guidelines for e-cigarettes as a smoking cessation aid. Examples of the data extraction form and detailed inclusion and exclusion criteria can be found in the published protocol paper [9]. The search strategy can be found in the Supplementary file (S2).

## Results

**Fig. 1:**
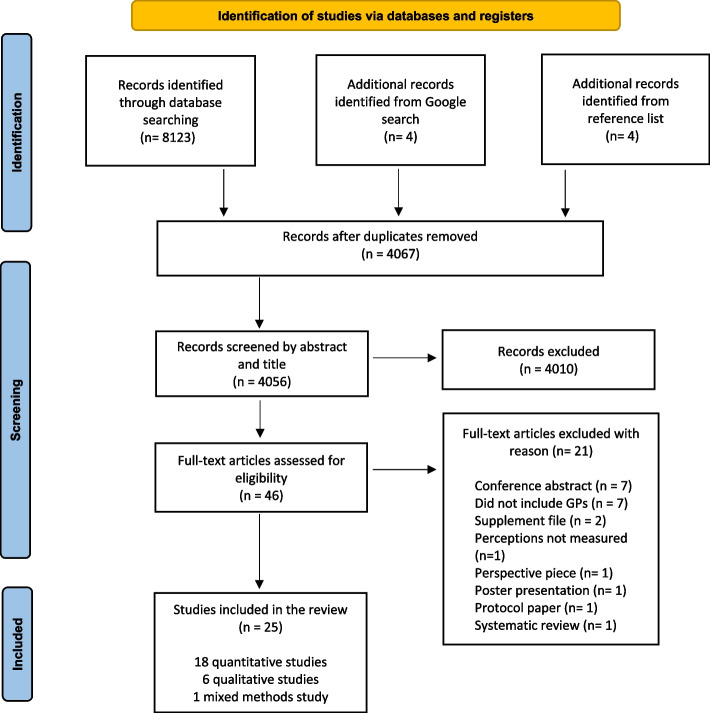
Methodological approach for systematic review and article selection (PRISMA diagram)

A total of 8123 studies were imported into Covidence for screening. After duplicates were removed there were 4056 studies screened by 2 authors for relevance by title and abstract. Following this, the full-text of *n* = 46 papers were read in full to determine eligibility and a further 21 studies were excluded leaving a final set of *n* = 25 articles (Fig. 1).

Of the 25 included articles, 18 were quantitative [12–29], 6 were qualitative [30–35] and 1 used mixed methods [36]. The majority of studies were conducted in the US [13, 15, 16, 20–23, 25, 26, 29–31, 33, 34, 36], four were conducted in the UK [12, 19, 32, 35] and one each from Belgium [27], China [14], Greece [18], Iran [24], Poland [28] and Slovenia [17].

Participants qualifications or medical specialities included family physicians/family medicine/family practice/general practitioners/general physicians/primary care physicians [12–36] (this group of participants will be referred to as GPs in this review) and GP trainees [19]. Many papers included findings from multiple specialities such as physician assistants [26, 30], nurses/nurse practitioners [17, 18, 22, 26, 30, 35], internal medicine [13, 15, 16, 20, 23, 31, 33], emergency medicine [13], preliminary and transitional medicine [13], medical professionals (undefined) [24], midwives [17, 32], obstetricians/gynaecologists [13, 16, 33, 34], cardiologists [13, 14, 18, 24, 25, 34], neurologists [13, 15], psychiatrists [15, 16], plastic surgeons [13], general surgeons [13, 15, 16, 20], pulmonologists/respiratory physicians [13, 15, 18, 20, 24, 25, 29, 34], pneumologists [14], allergists/immunologists [29], paediatricians [18, 21, 22, 24], internists [18, 24], ophthalmologists [13], anaesthesiologists [13, 20], otolaryngologists [13], orthopaedics [13], oncologists [12, 14, 25, 34], cancer surgeons [12], cancer nurse specialists [12], dentists [18], health visitors (trained nurse) [32] and stop smoking specialists/smoking cessation counsellors/tobacco counsellors [24, 27, 32].

Table 1 presents the characteristics of the quantitative studies and Table 2 those of the qualitative and mixed methods studies.

**Table 1:** Study characteristics of quantitative studies

Author/Year	Study population/ sample size	Country/city	Study Design	Gender	Age
Brett et al. (2020) [12]	**506 participants** GPs-103 Oncologists-102 Cancer surgeons- 100 Cancer nurse specialists-99	England, Wales, Scotland & Northern Ireland	Cross-sectional online survey. Face-validity. Questions developed from previous studies [15, 37, 38].	Male-41.1% Female-57.5% Prefer not to say- 1.4%	N/A
Egnot et al. (2017) [13]	**142 participants** (338 invited) 42% response rate Non-surgical clinicians- 102 (internal medicine, family medicine, preliminary and transitional medicine, cardiology, otolaryngology, emergency medicine, pulmonary critical care) Surgical clinicians- 40 (neurosurgery, plastic surgery, general surgery, obstetrics/gynaecology, anaesthesia, orthopaedics, ophthalmology)	Columbus, Ohio, USA	Cross-sectional online survey. Validity not established. Questions on harm reduction developed from previous studies [39].	Male- 69 Female- 73	Mean age- 28.95 years
Feng et al. (2019) [14]	**1023 participants** (1291 invited) Pneumology/cardiovascular/oncology- 190 Others- 833	China	Cross-sectional online survey. Validity not established. Questions developed from previous studies [20, 40].	Male- 692 Female-331	20–29 years- 226 30–39 years- 490 40 > years- 307
Kanchustambham et al. (2017) [15]	**115 participants** 40% response rate Internal medicine- 40 Internal medicine sub-specialities- 32 Family medicine- 10 Neurology- 5 Psychiatry- 7 Pulmonary- 14 Surgery- 7	Saint Louis University Hospital, Missouri, USA	Cross-sectional online survey. Validity not established. Questions developed from previous studies (not mentioned).	N/A	N/A
Kandra et al. (2014) [16]	**128 participants** (787 invited) 31% response rate Family medicine- 28 Internal medicine- 24 Obstetricians/gynaecologists- 21 Psychiatrists- 27 Surgeons- 28	North Carolina, USA	Cross-sectional online survey. Validity not mentioned.	N/A	< 44 years- 47.9% > 45 years- 52.1%
Koprivnikar et al. (2020) [17]	**479 participants** (1122 invited) Nurse or midwife- 356 Physician- 70 Other- 52	Slovenia	Cross-sectional online survey. Validity not established. Pilot tested. Questions developed from previous studies [15, 20, 34, 38, 41].	Male- 47 Female- 431	20–39 years- 235 (49.1%) > 40 years- 244 (50.9%)
Moysidou et al. (2016) [18]	**262 participants** (865 invited) 30.3% response rate Physician- 185 Nurse- 77 Physician specialties: GP- 17 Cardiologist- 53 Dentist- 32 Internist- 32 Paediatrician- 17 Respiratory physician- 34	Athens, Greece	Cross-sectional online survey. Validity not established.	Male- 138 Female- 124	Mean age- 39 years
Mughal et al. (2018) [19]	**312 participants** GPs- 117 GP trainees- 189	UK	Cross-sectional online survey. Pilot tested. Validity not established.	Male- 110 Female- 201	N/A
Nickels et al. (2017) [20]	**561 participants** (1500 invited) 44% response rate Anaesthesiology- 64 Family practice- 97 General surgery- 107 Internal medicine- 89 Pulmonary- 204	USA	Cross-sectional postal survey. Face-validity and cognitively tested.	Male- 464 Female- 97	Mean age- 57.2 years
Pepper et al. (2015) [21]	**776 participants** (2368 invited) 33% response rate Paediatrics- 410 Family medicine- 366	USA	Cross-sectional online survey. Pilot tested. Validity not mentioned	Male- 526 Female- 250	N/A
Pepper et al. (2014) [22]	**561 participants** (3923 invited) 28% response rate Family medicine- 258 Paediatrician- 114 Nurse practitioner- 189	Minnesota, USA	Cross-sectional online survey. Validity not mentioned. Cognitively tested. Questions developed from previous studies [42].	Male- 161 Female- 400	Mean age- 47.8 years
Salloum et al. (2021) [23]	**216 participants** (801 invited) 28.7% response rate Family medicine- 98 Internal medicine- 103 Missing- 15	Florida, USA	Cross-sectional state-wide postal survey. Questions guided and developed from previous studies [16, 36, 43]. Face-validity and cognitively tested.	Male- 163 Female- 46 Missing-7	Mean age- 58.6 years
Sharifi et al. (2019) [24]	**147 participants** Medical professionals, smoking cessation counsellors, respiratory physicians, cardiologists, internists, paediatricians & GPs	Iran	Cross-sectional postal survey. Questions developed from previous validated studies [44].	Male- 49 Female- 98	Mean age- 41.05 years
Steinberg et al. (2015) [25]	**158 participants** 2.25% response rate (pilot study) Primary care- 62 Specialists (tobacco diseases) oncologists, cardiologists, pulmonologists- 96	USA	Cross-sectional online survey. Validity not mentioned	Male- 44 Female-112 Missing-2	N/A
Talley et al. (2017) [26]	**80 participants** (181 invited) 44% response rate (pilot study) Physician- 48 Nurse- 8 Physicians’ assistant- 6 Nurse practitioner- 16 Other (general or family practice)- 2	USA	Cross-sectional online survey. Validity not mentioned.	Male- 41 Female- 39	Mean age- 45.6 years
Van Gucht & Baeyens (2016) [27]	**76 participants** GPs- 22 (29%) Tobacco counsellors- 54 (71%)	Flanders, Belgium	Cross-sectional online questionnaire. Some questions developed from previous studies [16]. Validity not mentioned.	Male: GPs- 33% Tobacco counsellors- 40% Female: GPs-67% Tobacco counsellors- 60%	Mean age- 45 years
Zgliczynski et al. (2019) [28]	**412 participants** (500 invited) 82.4% response rate Physicians	Poland	Cross-sectional postal survey. Face-validity. Questions developed from previous studies [16, 22, 27].	Male- 147 Female- 265	Mean age- 31.9 years
Zhou et al. (2020) [29]	**291 participants** 46% response rate Primary care physicians (internists, paediatricians or family medicine physicians)- 222 Pulmonary- 33 Allergists/immunologists- 36	Michigan, USA	Cross- sectional online survey. Questions developed from previously validated surveys [45, 46].	Male- 160 Female- 131	< 30 years- 125 31–40 years- 70 41–50 years- 42 51–60 years- 34 > 60 years- 20

**Table 2:** Study characteristics of qualitative studies

Author/Year	Study population/ sample size	Country/city	Study Design	Gender	Age
Bascombe et al. (2016) [30]	**20 interviews** Physicians-15 Nurses-3 Physician assistants-2 Georgia- 6 Atlanta- 60 invited, 14 interviewed	Atlanta metro and rural southern Georgia, USA	Semi-structured interviews.	Female-13 Male-7	Mean age- 45.25 years
El-Shahawy et al. (2016) [31]	**15 interviews** Family medicine physicians-11 Internal medicine physicians-4 University health system- 46 invited, 7 interviewed ACORN- 26 invited, 8 interviewed	Virginia, USA	Semi-structured interviews.	Female- 47% Male- 53%	Mean age- 43.1 years
Hunter et al. (2021) [32]	**60 interviews** Midwives-17 Health visitors-10 General practitioners-15 Stop smoking specialists-18	UK (including Wales and Northern Ireland)	In-depth interviews.	Female-50 Male-10	18–25 years- 3 26–35 years- 16 36–45 years- 13 46–55 years- 21 56–65 years- 6 66+ years- 1
Kollath-Cattano et al. (2019) [33]	**14 interviews** (18 expressed interest) Internal medicine-5 Family medicine-6 Obstretics/gynaecology-3	South Carolina, USA	Semi-structure interview.	N/A	N/A
Ofei-Dodoo et al. (2017) [36]	**117 family physicians** (154 invited)	Kansas, USA	Mixed methods. Questionnaire and in depth interviews	Female-50 Male-57	N/A
Singh et al. (2017) [34]	**35 interviews** Primary care physicians-10 Obstetrics/gynaecology-10 Cardiologists-5 Pulmonologists-5 Oncologists-5	USA	Semi-structured interview.	Female- 8 Male- 27	Mean age- 55.5 years
Stepney et al. (2019) [35]	**23 interviews** (45 invited) General practitioners-15 Nurses-8	Thames Valley and South Midlands, West of England, Eastern and East Midlands, UK	Semi-structured interviews.	N/A	N/A

### Synthesis of studies using a quantitative design

Findings from eighteen quantitative studies were included in the synthesis [12–29]. From the eighteen quantitative papers six articles used validated instruments [12, 20, 23, 24, 28, 29], three articles used instruments that were cognitively tested [20, 22, 23] and 11 articles adapted questions from other published studies [12–15, 17, 22, 23, 27–29, 44].

Data were explored to understand (1) knowledge, (2) attitudes and beliefs, (3) recommendations and, (4) comfort and confidence levels of GPs when discussing e-cigarettes with patients.

#### Perceived knowledge of e-cigarettes as a smoking cessation aid

Across the studies, GPs had limited knowledge of e-cigarettes however, they were reported as having greater knowledge of e-cigarettes than paediatricians, nurse practitioners and cancer surgeons [12, 18, 22]. GPs wanted to receive training and learn more about e-cigarettes for smoking cessation to better advise their patients on suitability of using e-cigarettes to quit smoking [12, 21, 22, 24, 26, 28].

Information regarding e-cigarettes was sought by GPs from government/health organisations, healthcare colleagues, news/media/advertisement, scientific literature, professional organisations, professional guidelines/development/training, charities, family members, friends and patients [12, 13, 22, 23, 28].

#### Attitudes and beliefs of GPs on e-cigarettes for smoking cessation

The majority of GPs surveyed in four studies [19, 26, 28, 29] believed e-cigarette use to be harmful, while other studies reported that GPs thought e-cigarettes were less harmful than regular cigarettes [18, 21, 22, 24]. In one study, 25% of respondents were unsure if e-cigarettes were less harmful than standard cigarettes [12] and in another, GPs somewhat agreed that e-cigarettes were safer than other tobacco products [22].

In other studies, GPs commented on the possibility that e-cigarettes could be a gateway to smoking especially amongst adolescents and non-smokers [15, 21, 22, 27, 28], and felt it was important to address this issue with patients [22].

There were mixed views amongst tobacco counsellors and GPs on the use of e-cigarettes as smoking cessation aids. In Belgium, tobacco counsellors ‘totally disagreed’ more than GPs (24% vs 5%) that e-cigarettes were an effective method to quit smoking [27].

In regards to whether e-cigarettes containing nicotine are addictive, in one study, 46% of GPs totally agreed compared to 56% of tobacco counsellors. Similarly, 18% of GPs totally disagreed that e-cigarettes without nicotine are addictive compared to 20% of tobacco counsellors [27].

#### GPs recommendations of e-cigarettes as a smoking cessation aid

The majority of participants in the studies included in this review did not agree that e-cigarettes should be recommended as a smoking cessation treatment [13, 17, 18, 23, 27–29, 33–36]. In saying that, one study did find that if patients asked about e-cigarettes, GPs, would recommend them regardless of the lack of research and evidence behind the effectiveness and safety of the product [25].

#### GPs comfort and confidence levels discussing e-cigarettes with patients

One study reported that under half of GPs (48.2%) felt confident in their level of knowledge and capability to respond to patient questions compared to pulmonologists (65.5%) and surgical care providers (18.5%) (general surgeons/anaesthesiologists) [20].

In regards to comfort levels, two studies reported that GPs had higher knowledge and awareness of e-cigarettes than nurses and paediatricians, and were more comfortable with patient’s e-cigarette inquiries [22, 29]. Furthermore, GPs reported being more comfortable offering e-cigarette advice compared to allergy physicians [29].

Pepper et al. [22] compared younger and older GPs and found that younger GPs, had greater awareness of e-cigarettes but were not considered to be more comfortable around patients discussing e-cigarettes. Moysidou et al. [18] did not report on the exact figures of how many GPs thought e-cigarettes should be available on prescription, rather this was reported across physicians as a whole. Physicians (43.8%) believed that e-cigarettes should only be available on prescription and 45.4% of them thought e-cigarettes should be licensed as medications [18]. Moreover, fewer than half of physicians (40.5%) believed that e-cigarettes should only be sold in pharmacies [18].

### Synthesis of studies using qualitative design

Findings from the six qualitative studies were included in the qualitative data synthesis [30–35] together with findings from the sole mixed method study [36]. Interview guides were used in all the studies and covered similar topics such as, beliefs, attitudes and perceptions, general knowledge, recommendations and screening and counselling practices for e-cigarettes. Studies assessed these issues in the context of the general population whereas Hunter et al. [32] was the only study that looked at these issues in the context of pregnancy and postpartum women. Kollath-Cattano et al. [33], was the only mixed method study and findings are included within the qualitative synthesis.

Interview questions reported in these studies asked about attitudes and beliefs of e-cigarettes as a smoking cessation aid such as, “what are your thoughts regarding e-cigarettes?” [31, 32, 34–36], “what are your perceptions on the safety and efficacy of e-cigarettes?” [32, 33, 36] and, “did you/have you recommended e-cigarettes to quit smoking?” [30–32, 36]. Only two studies asked about prescribing and licensing with “how do you feel about the licensing of e-cigarettes?” [32, 35].

Three themes were prominent among the included studies. These included (1) concerns, beliefs and lack of research, (2) recommendations of e-cigarettes as smoking cessation treatments and (3) e-cigarettes becoming an increasingly common topic in clinical consultations.

#### Theme 1: concerns, beliefs and lack of research on e-cigarettes as a smoking cessation aid

GPs held concerns around the safety and efficacy of e-cigarettes as a smoking cessation aid and believed there to be insufficient data on these issues [31, 34, 36]. They were also apprehensive about the health implications, long-term health risks that e-cigarettes may present and believed e-cigarettes are not completely risk free [30, 32].

“I tell them [patients] the jury is still out. We do not know about the long-term safety, we do not know about the efficacy [of e-cigarettes]” (primary care) [34].

GPs held concerns that e-cigarettes were not suitable for smoking cessation as they “still contained nicotine” and there remains a lack of evidence and knowledge to claim e-cigarettes as an effective method to quit smoking [36].

“There is not significant evidence showing e-cigarettes are effective in assisting in tobacco cessation.” (family physician) [36].

All seven studies identified a need for further research and information on e-cigarettes to support their clinical practice and ability to advise patients [30–36].

GPs indicated the lack of reliable scientific evidence about the safety of e-cigarettes as a major concern. They required more information on the long-term health effects before supporting and recommending the use of e-cigarettes as a smoking cessation treatment [30–36].

GPs held concerns about the increasing use and popularity of e-cigarettes amongst young people, adolescents and non-smokers as e-cigarettes were becoming a gateway to smoking for many of their patients in the US and UK [34, 35]. Many believed e-cigarettes to be a gateway for non-smokers to smoke combustible cigarettes and other tobacco products and for smokers, there were concerns around dual use [32].

“I am most concerned about gateway to other tobacco products and also impact on minors. I think that’s a big one and I’m very, very concerned about that.” (primary care) [34].

#### Theme 2: GP recommendations of e-cigarettes as smoking cessation treatments

GPs had mixed views recommending e-cigarettes for smoking cessation. Most were reluctant to recommend e-cigarettes due to insufficient research around the safety and long-term health effects [32, 33, 36]. GPs were adamant the only way they would recommend or prescribe e-cigarettes for smoking cessation and take responsibility for their action, is if it has been approved by a regulatory authority [33, 35, 36].

“[E-cigarettes are] not regulated by [the] FDA and this seems to be a dangerous thing with possible carcinogenic effects.” (family physician) [36].

“Unless you show me some real evidence and the United States Preventative Task Force recommends it, I am probably not going to [recommend e-cigarettes].” (family medicine) [33].

#### Theme 3: e-cigarettes becoming increasingly common in clinical consultation

As e-cigarettes are becoming more common and readily available, smokers are increasingly seeking information and advice from their GPs on the potential harms and benefits for using e-cigarettes, including their possible role in supporting quit attempts. Three studies described GPs perceptions that their patients were initiating discussions more and more often with their GPs in clinical visits about e-cigarettes [31, 33, 34].

“E-cigarettes have definitely been coming up in the last six months, maybe the last year. More patients are mentioning it as an alternative or something they are looking to instead of traditional smoking.” (primary care physician) [31].

On the contrary, in some GP practices discussions of e-cigarettes did not take place [30], and in practices that it did, GPs did not feel they had a place or role to help patients quit smoking as their patients knew more about e-cigarettes than they did [35].

## Discussion

This systematic review synthesised evidence from literature describing the knowledge, attitudes, beliefs and social norms of GPs utilising e-cigarettes as a smoking cessation aid. There is modest evidence that GPs and their equivalents internationally are increasingly optimistic about the role of e-cigarettes as a smoking cessation aid. However, our review-found that some GPs have limited knowledge about e-cigarettes which affected their ability to confidently and comfortably communicate with their patients when discussing e-cigarettes for smoking cessation. Most GPs believed they had insufficient knowledge around e-cigarettes and lacked information they needed to confidently provide advice and guidance to their patients on e-cigarettes as a smoking cessation aid. Qualitative findings highlighted patients are increasingly seeking information and advice about e-cigarettes from primary care providers highlighting the need to address this knowledge gap amongst general practitioners.

Our review focusing specifically on GPs highlights mixed views regarding the safety and efficacy of e-cigarettes as smoking cessation aids and concerns about the potential of e-cigarettes becoming a gateway to tobacco smoking. A previous systematic review [47] described beliefs of a broad range of healthcare professionals (HCPs) regarding e-cigarette use for smoking cessation but did not provide comparison between different medical disciplines nor did it focus solely on GPs. Our review was able to go some way to defining the concerns of GPs specifically and how these differ to HCPs from different medical specialities.

The studies found consistent evidence that GPs, similar to other HCPs, felt they did not know enough about the use of e-cigarettes for smoking cessation and would like to learn more. GPs believed they had insufficient knowledge around e-cigarettes and rated themselves as having moderate to low levels of knowledge to provide information to their patients on e-cigarettes to help patients cease smoking [12–15, 17, 18, 28, 29].

Overall, GPs held diverse views on whether or not the risk of using e-cigarettes may cause certain types of diseases or illnesses for patients in the future. Some, GPs thought that e-cigarettes may increase the risk of cancer, cardiovascular and chronic lung diseases [27, 28] and others believed e-cigarettes to be less harmful than traditional cigarettes and safer than other tobacco products [18, 21, 22, 24], that e-cigarettes could help smokers quit smoking [24, 26, 29], that e-cigarettes had the likelihood of reducing the number of cigarettes smoked and saw them as a harm reduction tool [14, 23].

One common concern amongst GPs was that e-cigarettes may be a gateway to smoking. They suggested this issue be raised with patients and parents of adolescents [22] which might influence adolescents and non-smokers to take up smoking [14, 15, 22, 28]. This is consistent with emerging evidence from other studies such as research from Australia found that the use of e-cigarettes was three times higher among never smokers taking up smoking tobacco cigarettes, especially among adolescents [48]. A further study not only confirmed adolescent non-smokers that use of e-cigarettes had increased odds of smoking traditional cigarettes, but that cigarette smokers who had never used e-cigarettes were also more likely to take up e-cigarettes later in life [49].

We found that GPs who have sufficient knowledge on e-cigarettes were most likely to perceive e-cigarettes as a useful smoking cessation aid [13, 15, 21], and believe that e-cigarettes could reduce cigarette consumption [20] among smokers that have failed to quit smoking through using alternative cessation methods and have refused to use approved medications [18, 23]. GPs that agree e-cigarettes are less harmful and safer than regular cigarettes [16, 21] were most likely to recommend e-cigarettes for smoking cessation. This could reflect the pragmatic, patient-centred focus of GPs and desire to work with patients to reduce harm, as a pathway to smoking cessation.

Overall, from the 25 articles that were included in this systematic review the vast majority of HCPs which included GPs in these reports were not confident in their abilities to have discussions or counsel patients on e-cigarettes and were not confident in their level of knowledge to answer patient queries regarding e-cigarettes [20, 23]. Eleven studies did not find HCPs would recommend e-cigarettes for smoking cessation [13, 17, 18, 23, 27–29, 33–36], nine studies recommended them [14–16, 19–21, 24, 25, 31], one remained cautious [12], one reported participants as having mixed views [32] and, three did not mention recommendations as this was not surveyed in the studies [22, 26, 30].

Variations in recommendations could be driven by different policies and guidelines applied in different countries and the diverse sources of information GPs access to learn about e-cigarettes. The social and professional norms within the workplace/health care setting with respect to smoking cessation may also impact their behaviours and recommendations to patients. For example, GPs that see patients in areas that have a higher uptake in smoking or where smoking is more of an issue could be more likely to recommend e-cigarettes as smoking cessation aids, than those who do not have patients that are smokers. Furthermore, some GPs were reluctant to recommend e-cigarettes as smoking cessation aids due to the lack of evidence, knowledge and research behind the safety and efficacy of these devices and as the evidence base grows their recommendations may change.

### Strengths and limitations

This is the first mixed-methods systematic review on GPs knowledge, attitudes, beliefs and prescribing intentions on e-cigarettes as a smoking cessation aid.

A limitation of the review is that findings were drawn from studies that included a number of different HCPs with GPs often just one speciality amongst HCPs from a range of specialities. As such, in some studies it was unclear which findings were applicable to the GP group. Some studies reported collecting data from ‘other’ HCPs that were not defined [14, 17, 26]. Similar to another earlier review on this topic, Erku et al. [47], was not able to identify any studies from low-middle income countries (LMICs) and therefore, the results from this review cannot be generalised to GPs globally. More studies are needed in LMICs where vaping is becoming more appealing to younger individuals. A meta-analysis was not conducted as most of the studies differ in terms of methodological approach, study aims and objectives, and survey items. Furthermore, due to the variations in questions on the perceptions of e-cigarettes for smoking cessation, it is difficult to compare these findings within the studies. Future research that is theoretically driven and focuses on GP perspectives will be valuable as GPs will continue to engage with e-cigarette use in communities with high uptake when discussing smoking cessation with their patients.

## Conclusion

GPs held mixed views on e-cigarettes as a smoking cessation aid. Some GPs were optimistic and had recommended e-cigarettes to their patients; others were reluctant and disagreed that e-cigarettes were an effective method to quit smoking. Most GPs lacked confidence and felt uncomfortable in having discussions with patients around e-cigarette safety and efficacy as a smoking cessation treatment. Limited knowledge about e-cigarettes is a concern, but in general GPs tended to have higher knowledge of e-cigarettes when compared to other HCPs. The literature supports the need for more information and training for GPs regarding e-cigarettes and in particular their potential role to support smoking cessation. Due to the availability and popularity of e-cigarettes, GPs are presented with challenges in supporting patients to quit smoking. Clear guidance on the role of e-cigarettes is needed to inform and upskill GPs about e-cigarettes as a smoking cessation aid. Further training and information is desired by GPs to enable them to discuss e-cigarettes and to confidently and comfortably answer patient concerns around e-cigarettes to quit smoking. This review will be useful to guide policy and contribute to guideline development that informs the potential role and place of e-cigarettes as a smoking cessation alternative.

## Supplementary Information

**Additional file 1: Supplementary file S1.** MMAT quality of studies.

**Additional file 2: Supplementary file S2.** Search strategy.

## Data Availability

The datasets used and/or analysed during the current study are available from the corresponding author on reasonable request.

## Abbreviations

GPs: General practitioners
E-cigarettes: Electronic cigarettes
ENDS: Electronic nicotine delivery systems
PRISMA: Preferred Reporting Items for Systematic Review and Meta-Analysis
MMAT: Mixed Methods Appraisal Tool
HCPs: Healthcare professionals
LMICs: Low-middle income countries
