# Supplementary material for: General practitioners’ knowledge, attitudes, beliefs and practices surrounding the prescription of e-cigarettes for smoking cessation: a mixed-methods systematic review
Source: BMC Public Health. 2022 Dec 23;22:2415. doi: 10.1186/s12889-022-14696-3 (PMC9784030; doi:10.1186/s12889-022-14696-3)
Supplement: Supplementary file 1 — Additional file 1: Supplementary file S1. MMAT quality of studies. [file 12889_2022_14696_MOESM1_ESM.docx]

Supplementary file S1. MMAT quality of studies

|  | **Low** | **Medium** | **High** |
| --- | --- | --- | --- |
| Bascombe et al (2016) (30) |  |  |  |
| Brett et al (2020) (12) |  |  |  |
| Egnot et al (2017) (13) |  |  |  |
| El-Shahawy et al (2016) (31) |  |  |  |
| Feng et al (2019) (14) |  |  |  |
| Hunter et al (2021) (32) |  |  |  |
| Kanchustambham et  al (2017) (15) |  |  |  |
| Kandra et al (2014) (16) |  |  |  |
| Kollath-Cattano et al (2019) (33) |  |  |  |
| Koprivnikar et al (2020) (17) |  |  |  |
| Moysidou et al (2016) (18) |  |  |  |
| Mughal et al (2018) (19) |  |  |  |
| Nickels et al (2017) (20) |  |  |  |
| Ofei-Dodoo et al (2017) (36) |  |  |  |
| Pepper et al (2015) (21) |  |  |  |
| Pepper et al (2014) (22) |  |  |  |
| Salloum et al (2021) (23) |  |  |  |
| Sharifi et al (2019) (24) |  |  |  |
| Singh et al (2017) (34) |  |  |  |
| Steinberg et al (2015) (25) |  |  |  |
| Stepney et al (2019) (35) |  |  |  |
| Talley et al (2017) (26) |  |  |  |
| Van Gucht & Baeyens (2016) (27) |  |  |  |
| Zgliczynski et al (2019) (28) |  |  |  |
| Zhou et al (2020) (29) |  |  |  |
