# Supplementary material for: General practitioners’ knowledge, attitudes, beliefs and practices surrounding the prescription of e-cigarettes for smoking cessation: a mixed-methods systematic review
Source: BMC Public Health. 2022 Dec 23;22:2415. doi: 10.1186/s12889-022-14696-3 (PMC9784030; doi:10.1186/s12889-022-14696-3)
Supplement: Supplementary file 2 — Additional file 2: Supplementary file S2. Search strategy. [file 12889_2022_14696_MOESM2_ESM.docx]

S2. Search strategy

| 1 | intent*.mp |
| --- | --- |
| 2 | view*.mp |
| 3 | perception*.mp |
| 4 | perspective*.mp |
| 5 | interest*.mp |
| 6 | awareness.mp |
| 7 | opinion*.mp |
| 8 | (belief* or believ*).mp |
| 9 | attitude*.mp |
| 10 | thought.mp |
| 11 | knowledge.mp |
| 12 | behavio?r.mp |
| 13 | general practitioners/or physicians, family/ or physicians, primary care/ |
| 14 | doctor* or family physician* or general practi* or family practi* or family medicine.mp |
| 15 | electr* cigar*.mp |
| 16 | (e-cig* or e?cig* or e-liquid* or e?liquid*).mp |
| 17 | (vape* or vaping).mp |
| 18 | vaping.mp. or exp Vaping/ |
| 19 | electronic nicotine.mp |
| 20 | electronic nicotine delivery systems/ or ENDS.mp |
| 21 | smoking.mp. or exp Smoking/ |
| 22 | smoking devices.mp |
| 23 | tobacco cessation.mp. or exp "Tobacco Use Cessation"/ |
| 24 | smoking cessation.mp. or exp Smoking Cessation/ |
| 25 | (ceas* or cessat* or quit* or stop or stopping).mp |
